# Multi-antigen MVA-vectored SARS-CoV-2 vaccine, GEO-CM04S1, induces cross-protective immune responses to ancestral and Omicron variants

**DOI:** 10.3389/fimmu.2025.1694699

**Published:** 2025-11-11

**Authors:** Amany Elsharkawy, Shannon Stone, Anchala Guglani, Felix Wussow, JD Burleson, Mary Hauser, Arban Domi, Pratima Kumari, Todd R. Albrecht, Chinonye Dim, Mark Newman, Don J. Diamond, Sreenivasa Rao Oruganti, Mukesh Kumar

**Affiliations:** 1 Department of Biology, College of Arts and Sciences, Georgia State University, Atlanta, GA, United States; 2 Department of Hematology and Transplant Center, City of Hope National Medical Center, Duarte, CA, United States; 3 GeoVax, Inc., Atlanta, GA, United States

**Keywords:** COVID-19, vaccine, SARS-CoV-2, K18-hACE-2, B.1, XBB.1.5, T-cells

## Abstract

The design focus of the first-generation COVID-19 vaccines was on the use of the SARS-CoV-2 spike (S) protein as the primary vaccine immunogen to induce high levels of neutralizing antibodies. Efficacy was repeatedly disrupted due to the diminished neutralizing capacity of vaccine-induced antibodies against emerging variants. Vaccine candidate GEO-CM04S1 is based on the use of a modified vaccinia Ankara vector (MVA) that co-expresses S and nucleocapsid (N) antigens of the Wuhan-Hu-1 reference strain. It is designed to induce both antibody and T-cell responses to both S and N, with the goal of broadening immune response specificity and function. Herein, we characterized GEO-CM04S1 vaccine induced immune responses and efficacy against the ancestral Wuhan strain B.1 and the Omicron subvariant XBB.1.5 in K18-hACE-2 mouse model. We also tested experimental vaccine candidates that encode either S or N proteins alone and determined their relative levels and immunogenicity and contribution to efficacy. We demonstrated that immune responses induced by GEO-CM04S1 protects against weight loss, upper and lower respiratory tract infection, lung injury and excessive inflammation following intranasal challenge with B.1. We showed that only GEO-CM04S1 maintained full protective efficacy against the Omicron subvariant XBB.1.5. GEO-CM04S1 vaccination reduced viral replication without significant lung damage following XBB.1.5 infection. Despite full protection, no neutralizing antibodies were detected against XBB.1.5 in the sera of GEO-CM04S1-immunized animals, suggesting a critical role of T-cell responses. Using antibody-mediated depletion, we showed that depletion of CD20 cells or CD8^+^ T cells did not impact the vaccine protective efficacy whereas depletion of CD4^+^ T-cells diminished levels of efficacy. Collectively, our data demonstrate the full cross-variant protective immunity induced by GEO-CM04S1 and that CD4^+^ T-cell responses are a major effector element of vaccine protection.

## 1 Introduction

The response to the global COVID-19 pandemic by public health entities and the vaccine industry was unprecedented and highly successful (1, 2). The focus of the first-generation vaccines was on the use of the SARS-CoV-2 Spike (S) protein as the primary vaccine immunogen (3, 4). However, multiple limitations associated with this approach are now evident due to the emergence of variants, with notable mutations in the S protein sequence (5–7). Consequently, vaccine efficacy was impacted due to the diminished neutralizing capacity of vaccine-induced antibodies allowing reinfections over time (8–10).

Evaluation of immune responses in COVID-19 convalescent patients demonstrated numerous viral proteins to be highly immunogenic, with respect to T-cell responses, and the data indicate the S and the Nucleocapsid (N) proteins are immunodominant antigens (11, 12). Our working hypothesis is that the inclusion of multiple SARS-CoV-2 antigens has the potential to broaden functional immunity and mitigate the impact of variants on vaccine efficacy. With this idea, the multi-antigen GEO-CM04S1 (originally designated COH04S1) was designed and produced as a next generation, viral vectored experimental vaccine.

Modified vaccinia Ankara (MVA) is a highly attenuated, replication-deficient strain of vaccina virus that is suitable for use as an efficient vaccine viral vector system due to its well-established safety and large insert coding capacity. Several COVID-19 vaccines using the MVA vector expressing SARS-CoV-2 S protein were evaluated for their protective efficacy and immunogenicity against SARS-CoV-2 challenge (13–16). GEO-CM04S1 is an MVA-vectored vaccine that co-expresses S and N gene products of the Wuhan-Hu-1 reference strain (B.1) (17). GEO-CM04S1 was demonstrated to be highly immunogenic in mice and protected Syrian hamsters and non-human primates against upper and lower respiratory tract infection following SARS-CoV-2 challenge with B.1 and several major variants, including the Omicron BA.1 and BA.2.12.1 variants (18–21). However, vaccine efficacy against severe SARS-CoV-2 infection is yet to be determined and direct data demonstrating the immunologic effectors that contribute to protection is lacking. In particular, the relative contribution of vaccine-elicited antibodies, CD4^+^ and CD8^+^ T cells in protection against severe SARS-CoV-2 infection has not yet been determined.

The K18-hACE2 transgenic (hACE2) mouse model is a well-established model of lethal SARS-CoV-2 infection used to study vaccines. Mice develop a severe respiratory disease that majorly recapitulates severe COVID-19 symptoms in humans, including severe lung pathology and excessive inflammation (5). In this study, we tested the efficacy of GEO-CM0S41 and experimental vaccine candidates that encode for either S or N proteins against SARS-CoV-2 B.1 and XBB.1.5 infection in the K18-hACE2 mouse model. We evaluated viral burden in the upper and lower respiratory tract and assessed lung pathology and inflammation. Additionally, we determined the relative contribution of CD4^+^ T-cells, CD8^+^ T-cells and B-cells in protection against severe disease using *in vivo* depletion experiments.

## 2 Materials and methods

### 2.1 Experimental vaccines

GEO-CM04S1 is an MVA-vectored vaccine that encodes SARS-CoV-2 S and N proteins based on the Wuhan-Hu-1 reference strain. MVA-vectored vaccine encoding for both S and N (GEO-CM04S1, MVA-S/N), MVA-vectored vaccine encoding for S only (MVA-S), MVA-vectored vaccine encoding for N (MVA-N), and empty viral vector (MVA) were constructed and produced using established cell culture methods and sucrose gradient purified (17).

### 2.2 *In vivo* mouse experiments

Animal studies were carried out in accordance with the recommendations of Institutional Animal Care and Use Committees (IACUC). The protocols were approved by the Georgia State University IACUC (Protocol number A24003). Experiments involving infectious SARS-CoV-2 were performed in the Animal Biosafety Level 3 laboratory. Virus inoculations were performed under anesthesia that was induced and maintained with isoflurane. Six-week-old K18-hACE-2 mice were assigned, with equal number of males and females, to each challenge group. Mice were intramuscularly vaccinated with two doses 28 days apart with 10^7^ plaque-forming units (PFU) of MVA-S/N, MVA-S, MVA-N, empty viral vector (MVA), or phosphate-buffered saline (PBS). At day 56 post first vaccine dose, animals were challenged intranasally (25 μl/nare) with 10^5^ PFU of SARS-CoV-2 B.1 (NR-52281, BEI Resources) (22, 23) or SARS-CoV-2 XBB.1.5 (NR-59104, BEI Resources) (24) or PBS (Mock). Body weight and clinical symptoms were recorded daily for 14 days. Groups of mice were euthanized using isoflurane for serum, lung, and nasal turbinate tissue collection at day 3 and day 6 post-challenge.

### 2.3 Infectious virus quantification by plaque assay

Lungs, nasal turbinates, and brain tissues were harvested from animals and flash-frozen in 2-methyl butane. Tissues were weighed and homogenized in a Fisherbrand™ Bead Mill 24 Homogenizer (BD Pharmingen, Catalog# 15-340-163) according to the manufacturer’s instructions. Tissue homogenates were clarified by centrifugation at 10,000 rpm for 10 mins and stored at −80 °C until further use. Virus titers were measured in tissue homogenates by plaque assay using Vero E6-TMPRSS2-ACE2 cells as described previously (24).

### 2.4 RNA extraction and quantitative PCR

SARS-CoV-2 RNA copies were assessed through quantification of N gene by RT-qPCR. Briefly, about 30 mg of frozen tissue was pounded and lysed in 600 μl RLT RNA extraction buffer (Qiagen) with 0.1% β-mercaptoethanol (β-ME). Tissue lysates were loaded onto the QIAshredder homogenizer (Qiagen, Catalog# 79656) and RNA was extracted with the Qiagen RNeasy Plus Mini Kit (Catalog# 74136) according to the manufacturer’s protocol and resuspended in RNAse-free water (25). The RNA concentration was determined with NanoDrop One instrument. The cDNA was synthesized from a 1000 ng/μL RNA using the iScript™ cDNA Synthesis Kit (Bio-Rad). Next, 2 μL of diluted cDNA was used per RT-qPCR. Viral RNA levels were measured with primers and probes specific for the SARS-CoV-2 N gene (Integrated DNA Technologies, Catalog# 10006713) and SsoAdvanced universal probes supermix (BIO-RAD, Catalog# 1725284). The number of viral RNA copies was calculated by extrapolation from the standard curve and expressed per μg of total RNA (23, 26).

### 2.5 Immunohistochemistry

Infected tissues were collected and placed in 4% paraformaldehyde (PFA) for histopathologic analysis. Tissues were processed in O.C.T blocks (Fisher Healthcare, Catalog# 23-730-571), sectioned at 5 microns thickness, and stained with hematoxylin/eosin (abcam, Catalog# ab245880). Tissue sections were stained with anti-SARS-CoV-2 N protein monoclonal antibody (Cell signaling Technology (HL344), Catalog# 26369) and Goat anti-Rabbit IgG (H+L) Cross-Adsorbed Secondary Antibody, Alexa Fluor™ 555 (Thermo Fisher Scientific, Catalog# A-21428). Additionally, lung tissue sections were stained with CD45-Alexa Fluor^®^ 488 (cell signaling technology, Catalog# D3F8Q) and anti-Actin α-Smooth Muscle-Cy3™ antibody (Sigma, Catalog# C6198). Stained sections were mounted with antifade mounting medium with DAPI. Images were acquired with the Invitrogen™- EVOS™ M5000 Cell Imaging System (27, 28).

### 2.6 Luminex assay

Lung tissues collected at day 3 post infection were weighed and homogenized in a Fisherbrand™ Bead Mill 24 Homogenizer (Catalog# 15-340-163) according to the manufacturer’s instructions. Lung homogenates were analyzed for cytokines and chemokines using MILLIPLEX^®^ Mouse Cytokine/Chemokine Magnetic Bead Panel (Millipore, Catalog# MCYPMX25-MAG). We calculated the sample concentrations using the Belysa^®^ Immunoassay Curve Fitting Software (Millipore Sigma) (29, 30).

### 2.7 Ab Neutralization assay

NAb were measured by plaque reduction neutralization assay (PRNT) assay using ancestral SARS-CoV-2 BA.1 or XBB.1.5 variant. Vero E6-TMPRSS2-ACE2 cells (BEI NR-54970) were seeded in 6-well plates at 200,000 cells/well in M199 medium with Earle’s salts (10X) supplemented with 5% inactivated fetal bovine serum, buffered with 3% sodium bicarbonate and Penicillin-Streptomycin for 3 days to form monolayer. Serum samples were diluted at 1:10 and were further serially diluted 4-fold from 1:10 to 1:5120. Next, 60 μl of serially diluted serum was mixed in 96-well plates with an equal volume of 100 PFU of SARS-CoV-2. Serum/virus mixtures were incubated for 30 mins at 37 °C. After incubation, 100 μl of serum/virus mixture was transferred to monolayered cells and incubated for 1 h at 37 °C. After incubation, 2 ml of 1% low melting agarose media was added to each well and plates were incubated at 37 °C for 2 days. After two days, plates were overlayed with 2% neutral red in 1% low-melting agarose for visualizing plaque formation. The number of plaques were counted and recorded in each well (26, 31).

### 2.8 Cell depletion and flow cytometry

Animals were treated intraperitoneally with 100 μL of mAb anti-mouse CD20 (MB20-11, IgG2c; BioXcell), mAb anti-mouse CD4 (clone GK1.5, IgG2b; BioXcell), mAb anti-mouse CD8β (clone 53–5.8, IgG1; BioXcell) at a dose of 500 μg/mouse a day prior to prime vaccine dose, a day prior to booster vaccine dose, and a day prior to SARS-CoV-2 virus challenge. The GK1.5 monoclonal antibody reacts with the mouse CD4 and has been shown to compete with clones YTS 177 and YTS 191 for CD4 binding. The anti-mouse CD20 (MB20-11) antibody has been reported to deplete B cells in mice within 1 hour of treatment and depletion lasts up to 57 days. The anti-mouse CD8β (clone 53–5.8) antibody has been shown to deplete CD8^+^ T cells completely but not CD8^+^ CD11c^+^ dendritic cells. Mice receiving an intraperitoneal injection of 100 μL of PBS was used as a control (27, 32–34).

Depletion of CD4^+^ T cell, CD8^+^ T cell, and CD20 was confirmed using flow cytometry. Briefly, mice were injected intraperitoneally with 100 μL depleting antibodies at a dose of 500 μg/mouse or with 100 μL PBS as a control (n=3 per group). Mice were euthanized 24 hrs following treatment using isoflurane and spleens were collected. We generated spleen single-cell suspensions using the gentle MACS tissue dissociator (Miltenyi Biotec, Catalog# 130-093-235). We incubated the spleen single-cell suspensions with Fc Block antibody (BD Pharmingen) in BD FACS™ Pre-Sort Buffer (BD Pharmingen) for 10 min at room temperature before staining. We incubated the cells with antibodies against the following markers: FITC Rat Anti-Mouse CD45 (BDB553080), PE-Cy™7 Hamster Anti-Mouse CD3e (BD Pharmingen, Catalog# 552774), PE Rat Anti-Mouse CD4 (BD Pharmingen, Catalog# 553730), PerCP-Cy5.5 Rat Anti-Mouse CD8β (BD Pharmingen, Catalog# 567597), and APC Rat Anti-Mouse CD20 (eBioscience, Catalog# QCH6A7). We used all antibodies at a concentration of 1 μg/10^6^ cells in 100 µL of pre-sort buffer. We stained the cells for 30 mins on ice, then washed and fixed them in fixation Buffer (eBioscience, Catalog# 00-8222-49). We acquired flow cytometry data on a BD LSRFortessa™ Cell Analyzer and analyzed the data using the FlowJo software.

### 2.9 Statistical analysis

Statistical analyses were performed using Prism 10. One-way ANOVA followed by Tukey’s multiple comparison test were used for statistical evaluation. The Log-rank (Mantel–Cox) test was applied for survival analysis. The statistical tests are indicated in the figure legend.

## 3 Results

### 3.1 Efficacy of different vaccine candidates against B.1 SARS-CoV-2 infection

**Figure 1:**
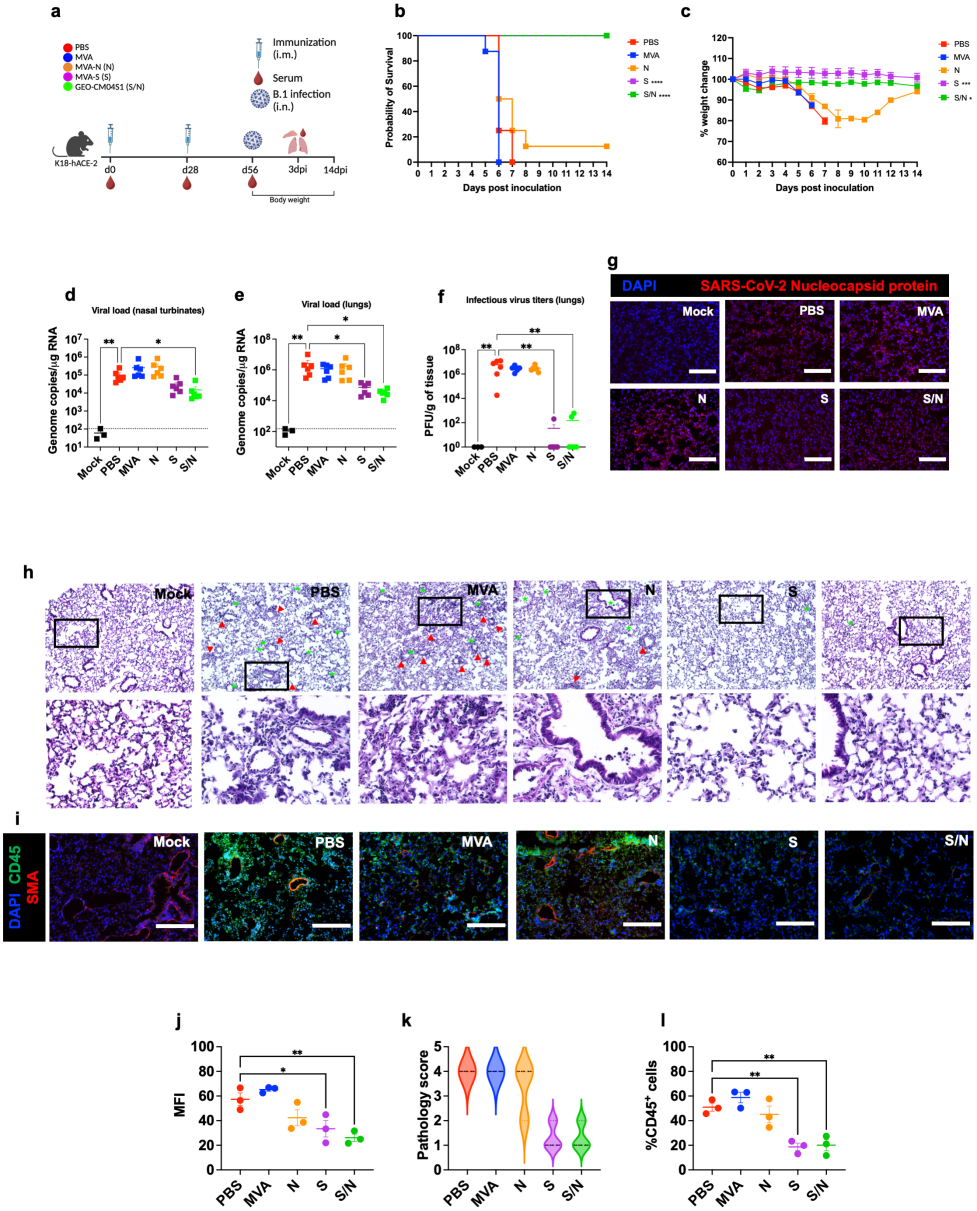
GEO-CM04S1 protects hACE2 mice against B.1 infection. **(a)** Scheme of the experiments. Mice (n = 14 per group; 7 males and 7 females) were immunized intramuscularly with 10^7^ PFU of MVA-only (MVA), MVA-N (N), MVA-S (S), or GEO-CM04S1 (S/N) in a prime-boost regimen. The control group received a placebo (PBS). Serum, lungs, and nasal turbinates were harvested at 3 dpi. **(b)** Kaplan–Meier survival curve for immunized mice following B.1 infection (n = 8; 4 males and 4 females per group). Statistical significance was determined by Log-rank Mantel-Cox test (****p < 0.0001). **(c)** Body weight change was monitored for 14 days post infection. Weights are expressed as the percentage of initial body weight. (n = 8; 4 males and 4 females per group). Statistical significance was determined by one-way ANOVA (*p < 0.05; ***p < 0.001). **(d, e)** Viral load in nasal turbinates and in the lungs (n = 6 per group; n = 3 for mock group) was quantified by RT-qPCR. The data are expressed on the log scale of the genomic copies/μg of RNA. Statistical significance determined by the Kruskal–Wallis test, followed by Dunn’s multiple comparisons test (*p < 0.05; **p < 0.01). **(f)** Infectious virus titers in the lungs were quantified by plaque assay (n = 6 per group; n = 3 for mock group). Statistical significance determined by a one-way ANOVA followed by Dunnett’s multiple comparisons (**p < 0.01). Each point represents an individual mouse. The bars indicate the mean ± SEM. **(g)** Lung samples were collected at 3 dpi and labeled with an antibody against the SARS-CoV-2 nucleocapsid protein. **(h)** H&E staining of lung tissue collected at 3 dpi. Perivascular cuffing (red triangle), leukocytes infiltration into alveolar space (green star). **(i)** Lung tissue collected at 3 dpi were stained with DAPI (blue), CD45 (green), and smooth muscle actin SMA (red). A representative image is shown for each group. Scale bar is 150μm. **(j)** Mean fluorescence intensity (MFI) was determined for lung sections stained for the SARS-CoV-2 nucleocapsid protein by ImageJ. **(k)** Lung pathology score is shown for lung sections (score 1: mild infiltration and alveolar thickening, score 2: moderate infiltration and alveolar thickening, score 3: severe infiltration and alveolar thickening score, score 4: very severe infiltration and alveolar thickening). **(l)** Percentage of CD45-positive cells in infected lung sections using ImageJ. Statistical significance determined by a one-way ANOVA followed by Dunnett’s multiple comparisons (*p < 0.05; **p < 0.01). The bars indicate the mean ± SEM.

Mice at 6-week-old were immunized with the PBS, MVA, MVA-S (S), MVA-N (N) or GEO-CM04S1 (N/S) as described and challenged intranasally with SARS-CoV-2 B.1 (10^5^ PFU/mouse). A group of PBS-immunized animals were challenged with PBS (mock-infected animals) (**Figure 1a**). Animals were monitored daily for illness signs, weight loss and survival for 14 days. PBS- and MVA-immunized animals succumbed to infection between 6–7 days post infection (dpi) (**Figure 1a**), documenting the lethality of the SARS-CoV-2 virus material and susceptibility of the mice. Both S- and S/N-immunized animals were fully protected against B.1 infection and did not lose significant weight or show any severe illness signs (**Figures 1b, c**). A slight protection was also observed in mice immunized with N vaccine showing 12.5% survival rate.

In independent experiments, mice were euthanized at 3 dpi and examined for viral titers. Using RT-qPCR, we measured the RNA viral load in the nasal turbinates and lungs. Compared to PBS group, only S/N-immunized animals showed significant reduction in the viral load in infected nasal turbinates (**Figure 1d**). In the lungs, both S- and S/N-immunized animals had significantly reduced viral load compared with the PBS group (**Figure 1e**). MVA- and N-immunized animals did not exhibit any significant reduction in viral load (**Figure 1e**). We next determined infectious virus titers in the lungs using plaque assay. Virus infectivity titers were significantly lower in both S- and S/N-immunized animals (**Figure 1f**). Consistent with the viral titer data, lung tissue samples from both S- and S/N-immunized animals showed reduced SARS-CoV-2 N protein signal. In contrast, infected lungs collected from PBS-, MVA- and N-immunized animals contained abundant SARS-CoV-2 N protein (**Figures 1g, j**).

We evaluated virus-induced lung injury through the histological analysis of tissue. Lungs collected from PBS-, MVA- and N-immunized animals revealed perivascular cuffing and alveolar space consolidation. Notably, the lungs from both S- and S/N-immunized animals displayed minimal pathology (**Figures 1h, k**). We also analyzed infected lung tissues for immune cell infiltration using CD45-specific antibody labeling. Consistent with histopathological analysis, lungs collected from PBS, MVA- and N-immunized animals revealed abundant CD45-positive immune cell infiltrates around the blood vessels and within the alveolar spaces. In contrast, both S- and S/N-immunized animals displayed low number of immune infiltrates into the lungs (**Figures 1i, l**).

**Figure 2:**
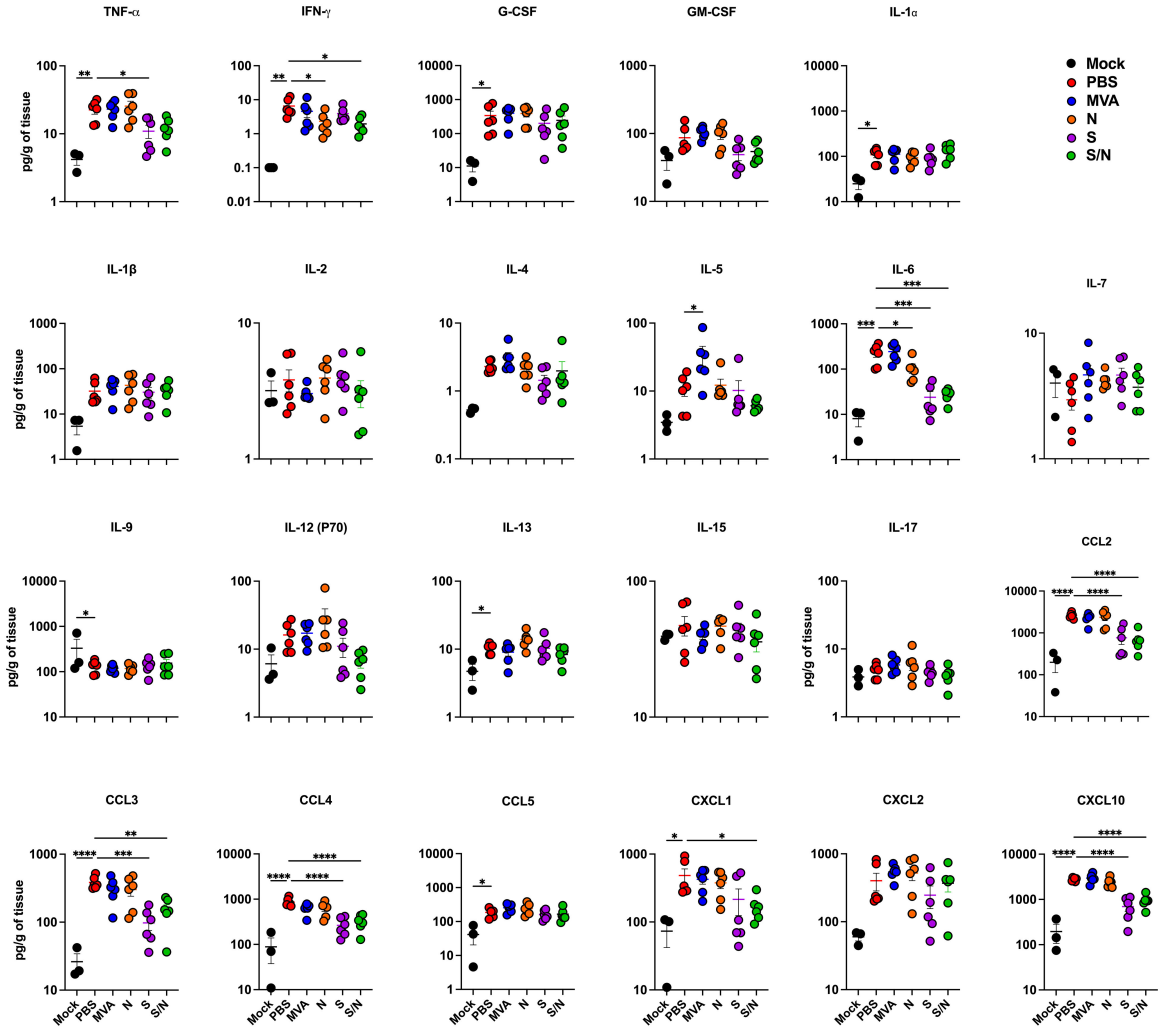
GEO-CM04S1 reduces lung inflammation following B.1 infection. Cytokine and chemokine protein levels in the lungs of immunized animals at 3 dpi following B.1 infection. The middle bar indicates the mean ± SEM (n = 6 per group; n = 3 for mock group). Statistical significance was determined by ordinary one-way ANOVA followed by Dunnett’s (*p < 0.05; **p < 0.01; ***p < 0.001, ****p < 0.0001). Each point represents an individual mouse. The bars indicate the mean ± SEM.

We next quantified the protein levels of proinflammatory cytokines and chemokines in the lungs using a multiplex immunoassay (**Figure 2**). Compared with PBS-immunized lungs, we detected significantly decreased levels of IFN-γ in N- and S/N-immunized lungs. We also detected decreased levels of IL-6 in infected lungs from N-, S- and S/N-immunized animals compared to PBS group. Moreover, only S- and S/N-immunized animals had reduced levels of CCL2, CCL3, CCL4 and CXCL10 chemokines after the infection compared to the PBS group. Only S/N-immunized animals had significantly decreased levels of CXCL1 in lungs compared to PBS-immunized animals.

### 3.2 GEO-CM04S1 maintains full protective efficacy against XBB.1.5 infection

**Figure 3:**
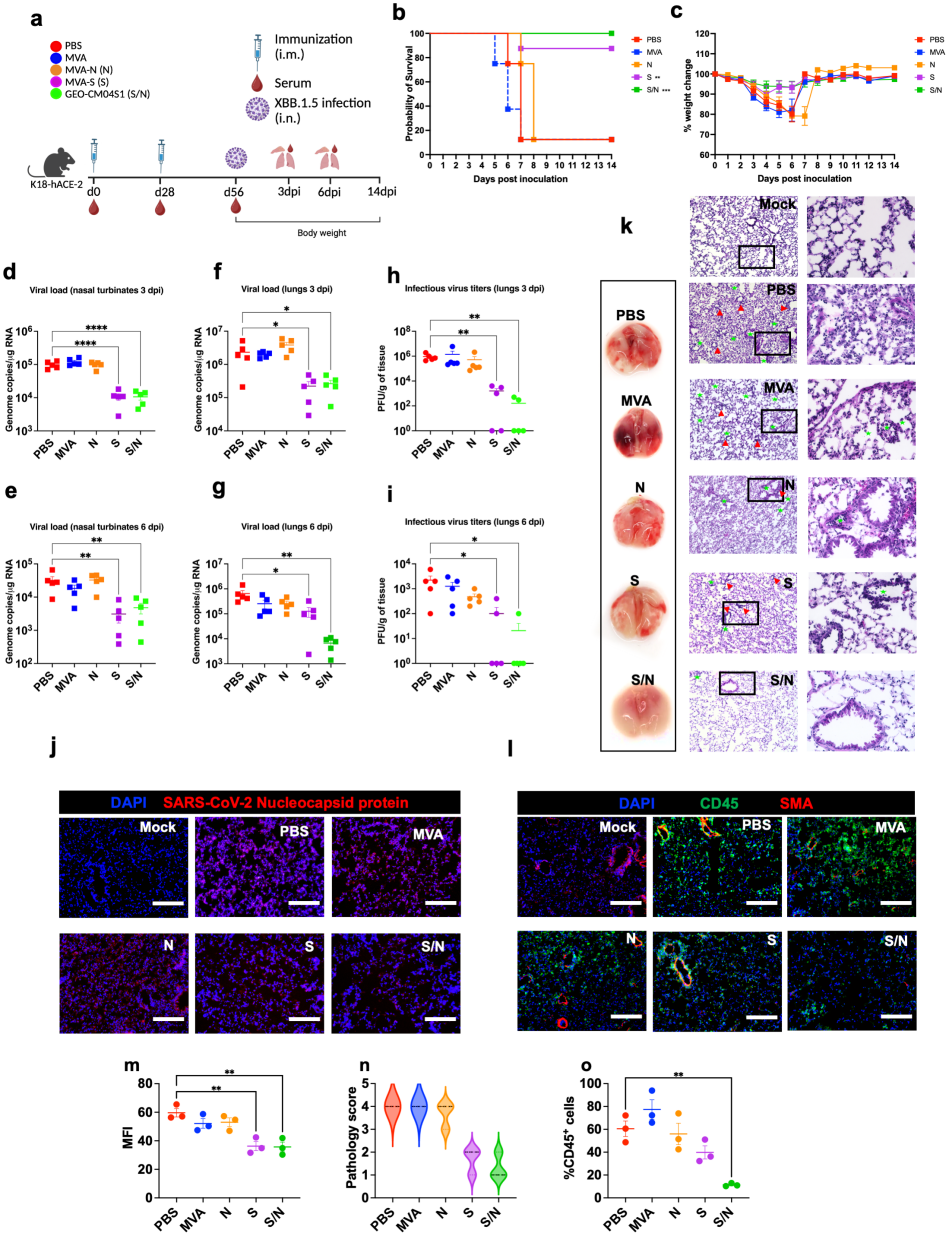
GEO-CM04S1 protects hACE2 mice against XBB.1.5 infection. **(a)** Scheme of the experiments. Mice (n = 18 per group; 9 males and 9 females) were immunized intramuscularly with 10^7^ PFU of MVA-only (MVA), MVA-N (N), MVA-S (S), or GEO-CM04S1 (S/N) in a prime-boost regimen. The control group received a placebo (PBS). Serum, lungs, and nasal turbinates were harvested at 3 and 6 dpi. **(b)** Kaplan–Meier survival curve for immunized mice following XBB.1.5 infection (n = 8; 4 males and 4 females per group). Statistical significance was determined by Log-rank Mantel-Cox test (**p < 0.01; ***p < 0.001). **(c)** Body weight change was monitored for 14 days post infection. Weights are expressed as the percentage of initial body weight. (n = 8; 4 males and 4 females per group). **(d, e)** Viral load in nasal turbinates (n = 5 per group) was quantified by RT-qPCR at 3 and 6 dpi. The data are expressed on the log scale of the genomic copies/μg of RNA. **(f, g)** Viral load in the lungs (n = 5 per group) was quantified by RT-qPCR at 3 and 6 dpi. The data are expressed on the log scale of the genomic copies/μg of RNA. **(h, i)** Infectious virus titers in the lungs were quantified by plaque assay at 3 and 6 dpi (n = 5 per group). Statistical significance determined by a one-way ANOVA followed by Dunnett’s or Bonferroni’s multiple comparisons test (*p < 0.05; **p < 0.01; ****p < 0.0001). Each point represents an individual mouse. The bars indicate the mean ± SEM. **(j)** Lung samples were collected at 3 dpi and labeled with an antibody against the SARS-CoV-2 nucleocapsid protein. **(k)** Gross lung pathology at 6 dpi and H&E staining of lung tissue collected at 3 dpi. Perivascular cuffing and leukocytes infiltration into alveolar space (red triangle). **(l)** Lung tissue collected at 3 dpi were stained with DAPI (blue), CD45 (green), and smooth muscle actin SMA (red). A representative image is shown for each group. Scale bar is 150μm. **(m)** MFI was determined for lung sections stained for the SARS-CoV-2 nucleocapsid protein by ImageJ. **(n)** Lung pathology score is shown for lung sections (score 1: mild infiltration and alveolar thickening, score 2: moderate infiltration and alveolar thickening, score 3: severe infiltration and alveolar thickening score, score 4: very severe infiltration and alveolar thickening). **(o)** Percentage of CD45-positive cells in infected lung sections using ImageJ. Statistical significance determined by a one-way ANOVA followed by Dunnett’s multiple comparisons (**p < 0.01). The bars indicate the mean ± SEM.

We evaluated the cross-protective efficacy of the vaccine candidates against the SARS-CoV-2 Omicron variant XBB.1.5. Mice at 6-week-old were immunized with the PBS, MVA, MVA-S (S), MVA-N (N) or GEO-CM04S1 (N/S) and challenged intranasally with SARS-CoV-2 XBB.1.5 (10^5^ PFU/mouse). We used the Omicron XBB.1.5 variant because it was the prevalent variant at the time of experiments. Animals were monitored daily for illness signs, weight loss and survival for 14 days (**Figure 3a**). Expectedly, PBS- and MVA-immunized animals succumbed to infection by 7 dpi. Notably, there was a delay in the development of illness signs in N-immunized animals, however they succumbed to infection by 8 dpi. Interestingly, S-immunized also experienced some weight loss and 10% animals in this group succumbed to the infection. Unlike S-immunized mice, S/N-immunized mice maintained full protective efficacy against XBB.1.5 infection (**Figures 3b, c**). The S/N-immunized mice did not lose significant weight or show any severe illness signs.

In independent experiments, infected mice were euthanized at 3 and 6 dpi and tissues were collected for further analysis. Using RT-qPCR, we measured the RNA viral load in the nasal turbinates and lungs. Compared to PBS group, both S- and S/N-immunized animals showed significant reduction in the viral load in nasal turbinates at both 3 and 6 dpi (**Figures 3d, e**). In the lungs, both S- and S/N-immunized animals had significantly reduced viral load compared with the PBS group. On the other hand, MVA- and N-immunization did not result in significant reduction in viral load (**Figures 3f, g**). Additionally, using plaque assay, we determined infectious virus titers in the lungs. Virus titers were significantly lower in both S- and S/N-immunized animals (**Figures 3h, i**).

Using immunofluorescence labeling, we analyzed infected lung tissues for antigen presence and immune cell infiltration. Consistent with the viral titer data, lung tissue samples from both S- and S/N-immunized animals showed reduced SARS-CoV-2 N protein signal. In contrast, infected lungs collected from PBS, MVA-, and N-immunized animals contained abundant SARS-CoV-2 N protein at 3 dpi (**Figures 3j, m**).

Next, we evaluated the virus-induced lung injury through the histological analysis of the lung tissue. Lungs collected from MVA- and N-immunized animals revealed perivascular cuffing and alveolar space consolidation at 3 dpi. Interestingly, the infected lungs from S-immunized animals also exhibited some pathological changes. Notably, the infected lungs from S/N-immunized animals displayed minimal pathology (**Figures 3k, n**). To assess immune cell infiltration into the lungs, we deployed CD45-specific antibody labeling. Consistent with histopathological analysis, lungs collected from PBS-, MVA-, and N-immunized animals revealed abundant CD45-positive immune cell infiltrates around the blood vessels and within the alveolar spaces. In contrast, both S- and S/N-immunized animals displayed low number of immune infiltrates into the lungs at 3 dpi (**Figures 3l, o**).

**Figure 4:**
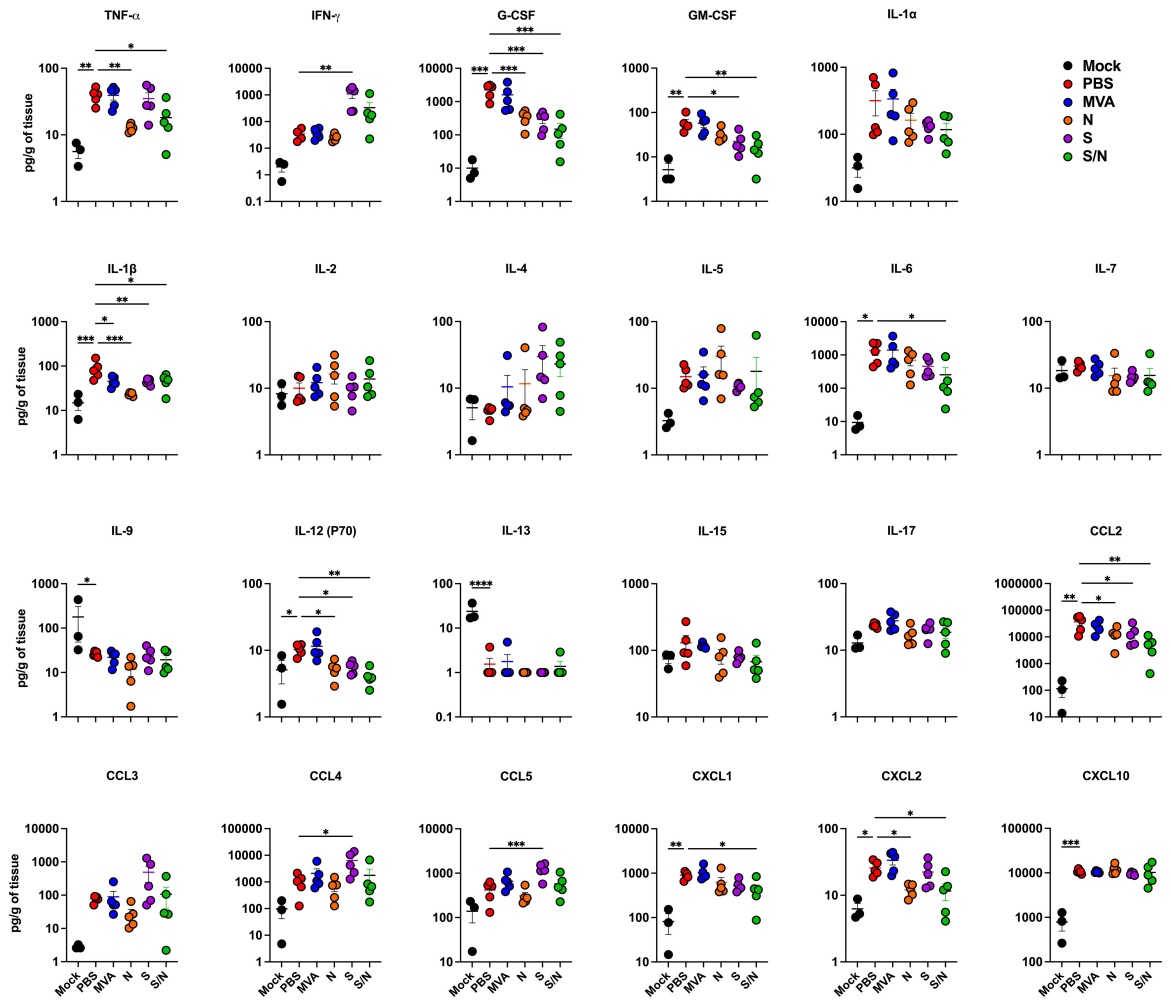
GEO-CM04S1 reduces inflammation following XBB.1.5 infection. Cytokine and chemokine protein levels in the lungs of immunized animals at 3 dpi following XBB.1.5. The middle bar indicates the mean ± SEM (*n* = 6 per group; *n* = 3 for mock group). Statistical significance was determined by ordinary one-way ANOVA followed by Dunnett’s (*p < 0.05; **p < 0.01; ***p < 0.001, ****p < 0.0001). Each point represents an individual mouse. The bars indicate the mean ± SEM.

Lung inflammation was further assessed by measuring the protein levels of proinflammatory cytokines and chemokines in infected lungs (**Figure 4**). Compared with PBS-immunized group, we detected significantly decreased levels of G-CSF, IL-1β, IL-12 (P70) and CCL2 in lungs from N-, S- and S/N-immunized animals. Notably, only N- and S/N-immunized animals showed significant decrease in several cytokines and chemokines including TNF-α and CXCL2. Only S/N-immunized had significant reduction in IL-6 and CXCL1 protein levels in infected lungs compared to the PBS group.

### 3.3 Neutralization capacity against SARS-CoV-2 B.1 and Omicron variants

**Figure 5:**
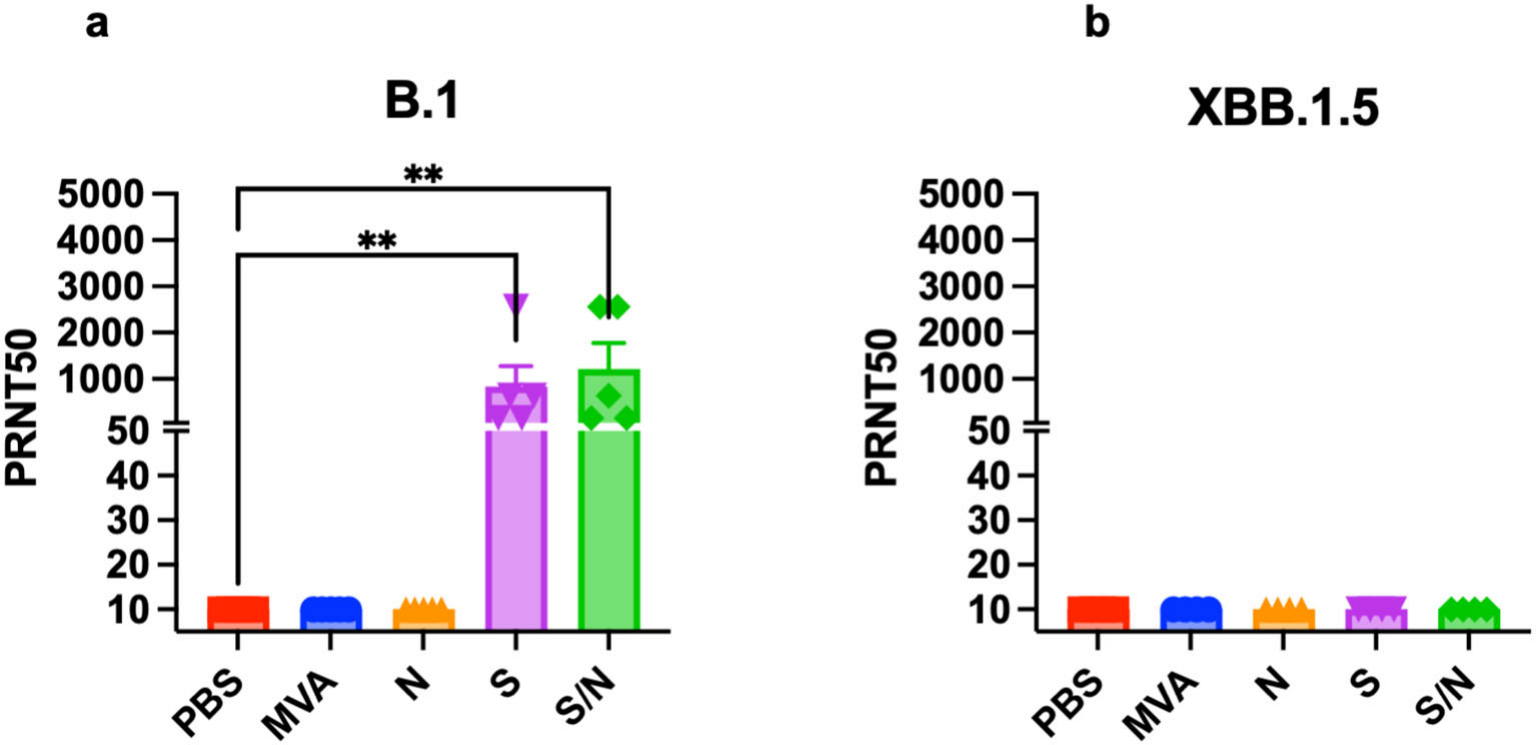
Serum SARS-CoV-2-specific neutralizing antibodies. Mice were vaccinated with two doses of PBS, MVA, N, S, or S/N, 28 days apart. Sera collected from vaccinated mice were analyzed for neutralization activity against **(a)** SARS-CoV-2 B.1 (n = 5) and against **(b)** Omicron subvariant XBB.1.5 (n = 4) via PRNT assay. Each point represents an individual mouse. Bar represents the mean ± SEM. Statistical significance was determined by Kruskal Wallis test, followed by Dunn’s test (**p < 0.01).

We analyzed the levels of SARS-CoV-2-neutralizing antibodies in sera collected from mice immunized with two doses of the, MVA, MVA-S, MVA-N or GEO-CM04S1 (S/N). Sera from both S- and S/N-immunized animals showed high levels of neutralizing antibodies against B.1 SARS-CoV-2 (**Figure 5a**). In contrast, no neutralizing antibodies were detected against XBB.1.5 in any of the groups (**Figure 5b**). These results suggest that vaccine-induced antibodies are not the major immunologic determinant of protection in this model.

### 3.4 CD4^+^ T-cells are the major immunologic determinant of GEO-CM04S1 protection

**Figure 6:**
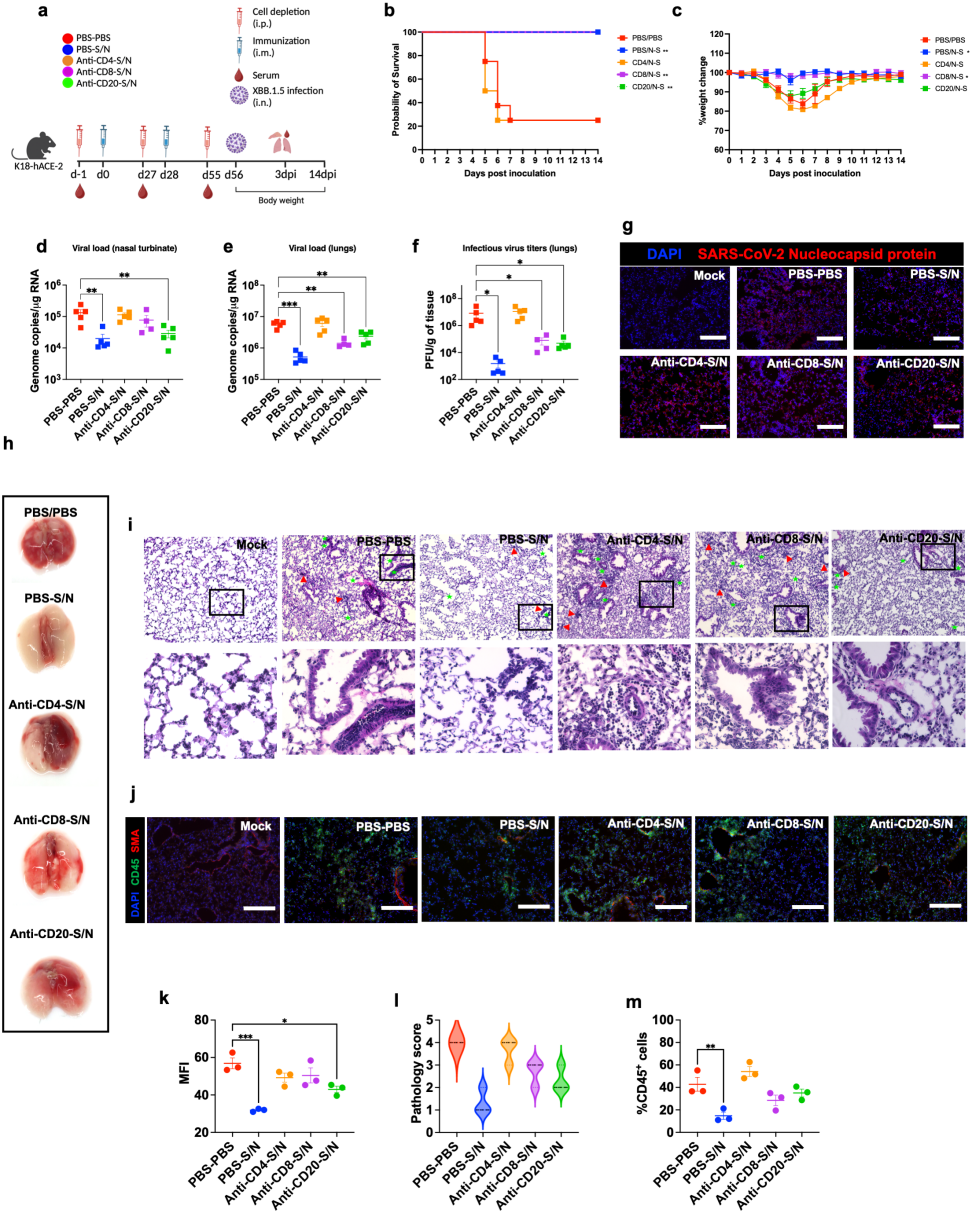
GEO-CM04S1–induced CD4^+^ T-cells provide protection against XBB.1.5 infection. **(a)** Scheme of the experiments. Mice (n = 12–13 per group) were treated intraperitoneally with Anti-CD4, Anti-CD8, Anti-CD20, or PBS a day prior to intramuscular immunization with 10^7^ PFU of GEO-CM04S1 (S/N) in a prime-boost regimen and a day prior to virus infection. Serum, lungs, and nasal turbinates were harvested at 3 dpi. **(b)** Kaplan–Meier survival curve for treated mice following XBB.1.5 infection (n = 8). Statistical significance was determined by Log-rank Mantel-Cox test (**p < 0.01). **(c)** Body weight change was monitored for 14 days post infection. Weights are expressed as the percentage of initial body weight (n = 8). Statistical significance was determined by one-way ANOVA (*p < 0.05). **(d, e)** Viral load in nasal turbinates and in the lungs (n = 4–5 per group) was quantified by RT-qPCR. The data are expressed on the log scale of the genomic copies/μg of RNA. Statistical significance determined by a one-way ANOVA followed by Dunnett’s multiple comparisons test (**p < 0.01; ***p < 0.001). **(f)** Infectious virus titers in the lungs were quantified by plaque assay (n = 4–5 per group). Statistical significance determined by the Kruskal–Wallis test (*p < 0.05). Each point represents an individual mouse. The bars indicate the mean ± SEM. **(g)** Lung samples were collected at 3 dpi and labeled with an antibody against the SARS-CoV-2 nucleocapsid protein. **(h)** Gross lung pathology at 14 dpi in surviving mice. **(i)** H&E staining of lung tissue collected at 3 dpi. Perivascular cuffing (red triangle), leukocytes infiltration into alveolar space (green star).**(j)** Lung tissue collected at 3 dpi were stained with DAPI (blue), CD45 (green), and smooth muscle actin SMA (red). A representative image is shown for each group. Scale bar is 150μm. **(k)** MFI was determined for lung sections stained for the SARS-CoV-2 nucleocapsid protein by ImageJ. **(l)** Lung pathology score is shown for lung sections (score 1: mild infiltration and alveolar thickening, score 2: moderate infiltration and alveolar thickening, score 3: severe infiltration and alveolar thickening score, score 4: very severe infiltration and alveolar thickening). **(m)** Percentage of CD45-positive cells in infected lung sections using ImageJ. Statistical significance determined by a one-way ANOVA followed by Dunnett’s multiple comparisons (*p < 0.05; **p < 0.01; ***p < 0.001). The bars indicate the mean ± SEM.

We deployed antibody-mediated depletion of CD20^+^, CD4^+^ T-cells, and CD8^+^ T-cells. Using flow cytometry, we first confirmed successful depletion of target cells in the spleen 24 hrs following intraperitoneal treatment with depleting antibodies (n=3) (**Supplementary Figures 1a–c**). Mice were intraperitonially injected with depleting antibodies or PBS as a control 24 hrs prior to the administration of vaccine doses and virus challenge. For survival studies, we had 5 groups (n=8 per group, 4 males and 4 females). These groups are PBS-treated-PBS-vaccinated (PBS-PBS), PBS-treated-S/N-vaccinated (PBS-S/N), anti-CD4-treated-S/N-vaccinated (Anti-CD4-S/N), anti-CD8-treated-S/N-vaccinated (Anti-CD8-S/N), and anti-CD20-treated-S/N-vaccinated (Anti-CD20-S/N). Animals were then challenged intranasally with SARS-CoV-2 XBB.1.5 (10^5^ PFU/mouse) (**Figure 6a**). Expectedly, mice that were treated with PBS and did not receive the vaccine (PBS-PBS group) lost significant body weight and succumbed to infection with 75% mortality rate by 6 dpi. In contrast, S/N-immunized animals treated with only PBS (PBS-S/N group) were fully protected against XBB.1.5 virus challenge. Interestingly, S/N-immunized animals treated with anti-CD4 (Anti-CD4-S/N group) had reduced protection against XBB.1.5 infection, with significant weight loss and 75% mortality rate. S/N-immunized animals treated with CD8-depleting antibodies (Anti-CD8-S/N group) showed 100% survival rate without any significant weight loss. S/N-immunized animals treated with CD20-depleting antibodies (Anti-CD20-S/N group) lost some weight but did not succumb to infection (**Figures 6b, c**).

We also measured the RNA viral load in the nasal turbinates and in the lungs of the mice euthanized at 3 dpi. Compared to PBS group, only S/N-immunized animals that received only PBS or CD20-depleting antibodies showed significant reduction in the viral load in infected nasal turbinates. In comparison, S/N-immunized animals that received CD4- or CD8-depleting antibodies had high viral load in the nasal turbinates (**Figure 6d**). In the lungs, S/N-immunized animals that received PBS, CD8-, or CD20-depleting antibodies had significantly reduced viral load compared with the PBS group. Notably, S/N-immunized animals that received CD4-depleting antibodies had significantly higher viral load in the lungs (**Figure 6e**). Similar differences were observed in the levels of infectious virus titers between the groups as determined by plaque assay (**Figure 6f**). In addition, lung tissue samples from S/N-immunized animals that received PBS showed lower SARS-CoV-2 N protein signal compared to the other groups (**Figures 6g, k**).

Gross lung pathology at 14 dpi in surviving mice is shown (**Figure 6h**). We assessed lung pathology using H&E staining of lung sections collected at 3 dpi. S/N-immunized animals that received CD4-depleting antibodies revealed severe lung pathology including perivascular cuffing and alveolar space consolidation like PBS-PBS group. Notably, despite surviving the challenge, S/N-immunized animals that received CD8- or CD20-depleting antibodies displayed some lung pathology (**Figures 6i, l**). To access immune cell infiltrates into the lungs, we performed CD45 staining. Consistent with H&E staining, S/N-immunized animals that received CD4-depleting antibodies exhibited abundant CD45-positive immune cell infiltrates around the blood vessels and within the alveolar spaces. S/N-immunized animals that received CD8- or CD20-depleting antibodies also displayed modest increase in CD45 infiltration into the lungs. In contrast, lungs from S/N-immunized animals that received PBS displayed minimal pathology and low number of immune infiltrates into the lungs (**Figures 6j, m**).

## 4 Discussion

In this study, we demonstrated that GEO-CM04S1 protects hACE2 mice from severe respiratory infections upon challenge with either the ancestral Wuhan strain B.1 or Omicron XBB.1.5. GEO-CM04S1-vaccinated mice had lower viral burden in the nasal turbinates and in the lungs following B.1 infection. GEO-CM04S1 vaccination further protected mice against excessive lung pathology and inflammation. We also showed that MVA-S vaccine had similar efficacy against B.1, where the S protein is matched. Notably, minor levels of protection were also observed in mice immunized with N vaccine alone. Importantly, our data revealed that only GEO-CM04S1 maintained full protective efficacy against Omicron subvariant XBB.1.5, indicating a role for immune responses elicited by the N antigen where the S protein is mismatched. Additionally, immunization with GEO-CM04S1 reduced viral replication without significant lung damage. We also showed that, despite the absence of vaccine-induced neutralizing antibodies, GEO-CM04S1 maintained full protective efficacy against XBB.1.5 infection. We further showed that vaccine-induced CD4^+^ T-cells are essential for the development of protective immunity in this model. These results highlight the value of the increased breath of responses generated using a multiantigen vaccine approach including both S and N antigens over vaccine approaches utilizing S alone.

SARS-CoV-2-specific humoral and cellular immune responses against multiple SARS-CoV-2 antigens are well documented in COVID-19 convalescent individuals. Recent studies have shown the immunodominance pattern of multiple epitopes, including N protein, as targets to CD4^+^ and CD8^+^ T cell responses. Similarly, S- and N-peptide responsive CD4^+^ T cells, with a robust IFNγ response, were detected in PBMCs of COVID-19 convalescent patients (35). Several CD4^+^ and CD8^+^ T cell targets in SARS-CoV-2 have been detected, suggesting that inclusion of additional SARS-CoV-2 epitopes such as N has the potential to induce broader immune response.

Recently, several COVID-19 vaccine employing the MVA vector to express the SARS-CoV-2 spike (S) or the receptor binding domain (RBD) were evaluated for their protective potential against SARS-CoV-2 (36–39). These reports showed that the MVA vector vaccine platform is compatible/enhanced when combined with other vaccine platforms such as DNA or mRNA SARS-CoV2 vaccines and it also showed it could induce mucosal immunity if it is delivered via IN compared to IM vaccinations. The MVA- SARS-CoV2 vaccines can be further enhanced by stabilizing the spike protein from SARS-CoV2. GEO-CM04S1 is a synthetic MVA (sMVA) vector co-expressing full-length S and N antigens. The inclusion of the N antigen is a key design distinction, aimed at eliciting broader and more durable immune responses. It was previously demonstrated that GEO-CM04S1 can induce robust SARS-CoV-2 antigen-specific humoral and cellular immunity in mice (17). Previous results demonstrated the potent efficacy of GEO-CM04S1 against SARS-CoV-2 ancestral virus, the Beta and the Delta variants (18) as well as Omicron subvariants BA.1 and BA.2.12.1. In the present study, we used a lethal mouse model instead of mild SARS-CoV-2 associated disease models such as hamsters and non-human primate models. We directly compared the efficacy of GEO-CM04S1, MVA-S, and MVA-N against the B.1 and XBB.1.5 Omicron subvariant in a lethal mouse model. Complementing previous observations, we demonstrated that GEO-CM04S1 protected mice from severe SARS-CoV-2 infection caused by the B.1 and XBB.1.5 Omicron strains.

While vaccine-induced antibodies are particularly important in protecting against SARS-CoV-2 infection, recent variants are resistant to neutralization by sera from individuals vaccinated with COVID-19 vaccines that target the S protein based on the Wuhan strain (6, 8, 9). An often-overlooked group within vaccination strategies is patients with specific medical limitations, particularly those who are partially immunocompromised. This population frequently exhibits reduced capacity to generate and sustain protective antibody responses following administration of first-generation mRNA vaccines, resulting in significant variability in vaccine efficacy. Affected individuals include patients with various malignancies, autoimmune disorders, organ transplants, or those undergoing dialysis, as well as potentially older adults (40–51). While approved vaccines have generally demonstrated safety profiles comparable to the general population in these groups, allowing for the administration of additional booster doses and conferring incremental benefit. These limitations highlight the need for improved vaccine designs that can elicit stronger and more durable immune protection in immunocompromised individuals.

It was previously reported that GEO-CM04S1 protected Syrian hamsters against Omicron BA.1 and BA.2.12.1 (21). Here, we directly compared the efficacy of GEO-CM04S1, MVA-S, MVA-N against the Omicron subvariant XBB.1.5 in a lethal mouse model. We showed that GEO-CM04S1 protected mice from severe SARS-CoV-2 infection caused by the XBB.1.5 variant. MVA-S did not maintain full cross protection against the XBB.1.5 variant. While MVA-N provided slight protection against B.1, it did not protect against XBB.1.5, but only delayed disease symptoms. Interestingly, despite full protection against XBB.1.5 infection, no neutralizing antibodies were detected in the sera of GEO-CM04S1-vaccinated animals, suggesting that antibody response may not be the major correlate of vaccine protection. These results underscore the potential advantage of multivalent antigen design in MVA-vectored platforms and highlight the importance of incorporating conserved internal antigens such as N to enhance the breadth of vaccine-induced immunity.

Several studies have reported that T-cell responses to SARS-CoV-2 are more durable than antibody responses and are essential for long-lasting immunity (52, 53). Importantly, it was shown that early, or pre-existing, induction of T-cell responses correlated with mild disease and accelerated viral clearance in patients. Additionally, unlike their significant impact on the neutralization capacity of antibodies, SARS-CoV-2 variants (B.1.1.7, B.1.351, P.1, and CAL.20C) have insignificant impacts on total SARS-CoV-2-specific CD4^+^ and CD8^+^ T-cell reactivity, suggesting that T-cell epitopes are not highly variable amongst variants (11, 54). Therefore, the capacity of the recombinant sMVA-CoV-2 vectors to induce S- and N-specific T-cell responses present a significant advantage in providing a broader and more durable immunity to SARS-CoV-2 (19). However, it is still unclear whether vaccine-induced T-cell responses alone can protect against severe SARS-CoV-2 infection. In the current study, using antibody-mediated depletion of specific cells, we showed that CD4^+^ T-cell depletion diminished the vaccine protective efficacy. CD4^+^ T-cell depletion resulted in diminished protection against viral replication in the upper and lower respiratory tract and significant lung pathology. However, depletion of CD8^+^ T-cells did not result in a significant weight loss or mortality but diminished protection against viral replication in the upper respiratory tract. Additionally, we showed that CD20 B-cells depletion did not significantly impact vaccine protection against viral replication in the upper or lower respiratory tract following XBB.1.5 infection. These results suggest that T cell immunity alone, particularly CD4^+^ T-cells, may provide protection against SARS-CoV-2. However, our study does not include direct functional analyses of antigen-specific CD4^+^ or CD8^+^ T cells.

In summary, this study demonstrated the efficacy of GEO-CM04S1 to provide cross-protective immunity against SARS-CoV-2 and its emerging variants, complementing prior studies in animal models with COH04S1. In addition, it showed that vaccine-induced T-cell responses alone without neutralizing antibodies can provide protection against severe SARS-CoV-2 infection, consistent with prior studies demonstrating protection by COH05S1 against Omicron subvariants BA.1 and BA.2.12.1 even in the absence of detectable neutralizing responses. Our observations further support GEO-CM04S1 clinical evaluation as a COVID-19 vaccine candidate for widespread use including, potentially, use in immunocompromised patients where first-generation vaccines have failed to provide optimal benefit.

## Data Availability

The original contributions presented in the study are included in the article/**Supplementary Material**. Further inquiries can be directed to the corresponding authors.

## Supplementary material

Supplementary Figure 1 **(a)** Gating strategy and FACS plots of CD4^+^ T-cells in splenic tissues of PBS- and Anti-CD4-treated mice. Mice were treated Anti-CD4 intraperitonially at a dose of 0.5mg/mouse. FACS plots are shown for each individual mouse (n=3 per group). **(b)** Gating strategy and FACS plots of CD8^+^ T-cells in splenic tissues of PBS- and Anti-CD8-treated mice. Mice were treated Anti-CD8 intraperitonially at a dose of 0.5mg/mouse. FACS plots are shown for each individual mouse (n=3 per group). **(c)** Gating strategy and FACS plots of CD20^+^ B cells in splenic tissues of PBS- and Anti-CD20-treated mice. Mice were treated Anti-CD20 intraperitonially at a dose of 0.5mg/mouse. FACS plots are shown for each individual mouse (n=3 per group).
