# Supplementary figures and images for: Multi-antigen MVA-vectored SARS-CoV-2 vaccine, GEO-CM04S1, induces cross-protective immune responses to ancestral and Omicron variants
Source: Front Immunol. 2025 Nov 11;16:1694699. doi: 10.3389/fimmu.2025.1694699 (PMC12645453; doi:10.3389/fimmu.2025.1694699)

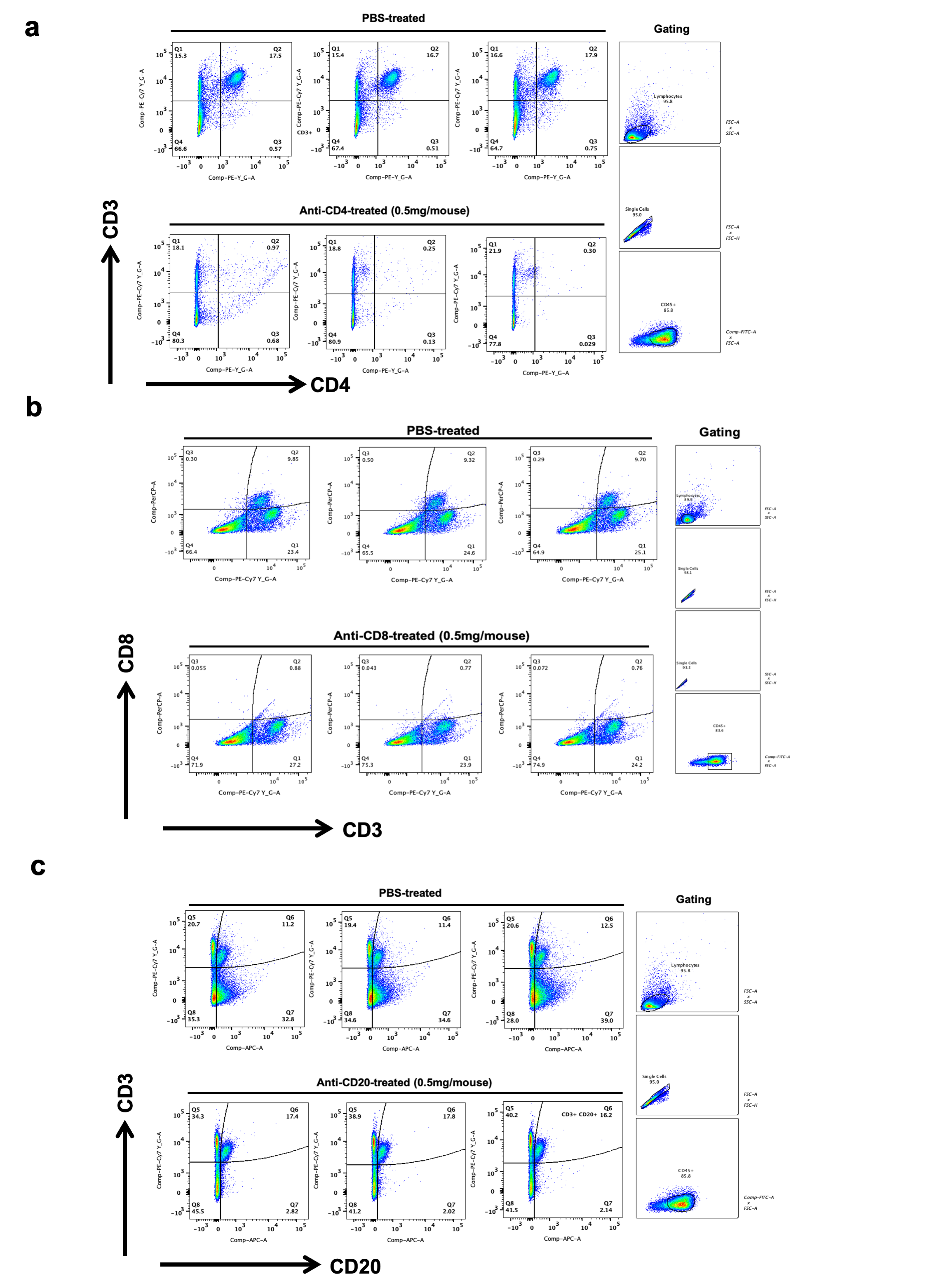

Supplement: Supplementary Figure 1 — (a) Gating strategy and FACS plots of CD4+ T-cells in splenic tissues of PBS- and Anti-CD4-treated mice. Mice were treated Anti-CD4 intraperitonially at a dose of 0.5mg/mouse. FACS plots are shown for each individual mouse (n=3 per group). (b) Gating strategy and FACS plots of CD8+ T-cells in splenic tissues of PBS- and Anti-CD8-treated mice. Mice were treated Anti-CD8 intraperitonially at a dose of 0.5mg/mouse. FACS plots are shown for each individual mouse (n=3 per group). (c) Gating strategy and FACS plots of CD20+ B cells in splenic tissues of PBS- and Anti-CD20-treated mice. Mice were treated Anti-CD20 intraperitonially at a dose of 0.5mg/mouse. FACS plots are shown for each individual mouse (n=3 per group). [file Image1.tiff]
